# Magnetic Resonance Imaging in the Evaluation of the Stress System in Acute and Chronic Cardiac Disease

**DOI:** 10.3390/diagnostics15131712

**Published:** 2025-07-04

**Authors:** George Markousis-Mavrogenis, Flora Bacopoulou, George Chrousos, Sophie I. Mavrogeni

**Affiliations:** 1 University Research Institute of Maternal and Child Health and Precision Medicine, and UNESCO Chair on Adolescent Health Care, National and Kapodistrian University of Athens Medical School, ‘Aghia Sophia’ Children’s Hospital, 11527 Athens, Greece; georgemm32@gmail.com (G.M.-M.); bacopouf@hotmail.com (F.B.); chrousos@gmail.com (G.C.); 2 Department of Cardiology, Angiology and Pneumology, Heidelberg University, 69120 Heidelberg, Germany; 3 German Centre for Cardiovascular Research (DZHK), Partner Site Heidelberg/Mannheim, 69121 Heidelberg, Germany

**Keywords:** heart failure, brain, immune system, stress system, magnetic resonance imaging

## Abstract

Various cardiac pathologies such as ischemic/non-ischemic heart disease, valvular heart disease and genetic heart disease may impair cardiac function and lead to heart failure (HF). Each individual condition but also the common endpoint of HF may involve the brain and the immune system next to the heart. The interaction of these systems plays an important role, particularly in the pathogenesis and prognosis of HF, and stress plays a pivotal role in this interaction. The stress system (SS) of the body can be activated by any stress factor exceeding a predefined threshold and all body structures including brain, heart and immune system can be affected. The SS is also responsible for body homeostasis. Both acute and chronic stress may lead to the development of acute and chronic heart disease. Magnetic Resonance Imaging (MRI) is the ideal noninvasive tool without radiation that can provide valuable information about the effect of the SS in various systems/organs using targeted protocols. A holistic approach provided by MRI has the potential to improve our knowledge regarding stress mechanisms on the axis of heart–brain–immune system in HF that may impact effective, individualized treatment. In this review paper, we describe how MRI can be used as a noninvasive tool to assess the effect of stress on the brain–immune system-heart-axis, discussing current possibilities, limitations and future directions.

## 1. Introduction

### 1.1. Brain–Heart–Immune Interaction in Acute/Chronic Cardiac Disease and Heart Failure

Various cardiac pathologies such as ischemic/non-ischemic heart disease, valvular heart disease and genetic heart disease may impair cardiac function and lead to the development of heart failure (HF). A mounting amount of evidence suggests that not only each individual condition but also the common endpoint of HF may involve the brain and the immune system next to the heart. The interaction of these systems plays an important role, particularly in the pathogenesis and prognosis of HF [1]. Intact cognitive function plays an important role in the various self-care activities and exerts a major impact on medication management, functional independence and life expectancy/prognosis in acute/chronic cardiac disease and HF [2].

Stress, either acute or chronic, plays an important role in both the development and evolution of various cardiac conditions. Takotsubo Syndrome (TTS) represents the best example of this close interaction [3]. The Comorbidity Frequency in the Takotsubo Syndrome (COUNTS) study indicated that emotional and physical stress were the respective triggers in 39% and 35% of 1109 TTS cases [3]. Furthermore, 24% of affected patients had known mental disorders, 7% showed nervous system disease with subarachnoid hemorrhage being the commonest cause and 1% had experienced trauma [3].

Taking the aforementioned evidence into account, a new paradigm of the brain-immune system-heart-axis is now coming into focus. This novel frontier is still not widely developed and will require an interdisciplinary collaboration in order for scientific understanding to increase so that clinical applications that can influence patient management can be developed. In this manuscript we attempt to summarize current evidence on the existence of a brain-immune system-heart axis and provide particular emphasis on the evaluation of the stress and immune systems using clinically employed and advanced magnetic resonance imaging applications.

### 1.2. Definition of Stress System

The stress system (SS) includes the Hypothalamic–Pituitary–Adrenal (HPA) axis, the brainstem Locus Coeruleus/Norepinephrine–Autonomic Nervous System (LC/NEANS) and the peripheral limbs of the latter, the sympathetic and parasympathetic nervous systems [4]. The SS is activated by any stress factor exceeding a predefined threshold and is also responsible for body homeostasis.

The response to stress includes the interaction of the central nervous system (CNS) and peripheral effectors through the secretion of the neurohormonal mediators, mainly corticotropin-releasing hormone (CRH), arginine-vasopressin (AVP) and norepinephrine in the brain, as well as glucocorticoids, norepinephrine, epinephrine, CRH and interleukin-6 (IL-6) in the periphery [4]. Central and peripheral effectors of the SS may influence the emotional/cognitive function of the neuro-cardio-metabolic and immunologic response of the body [4].

The stress response of the CNS can guide acute, time-limited adaptive mechanisms, such as the fight, flight or freeze reaction, that are responsible for acute, non-adaptive functions, such as reproduction, feeding and growth [5]. The adaptive response to stress can be either specific or relatively nonspecific to a stressor [6]. The inability of the SS to adequately control the adaptive response of the body may impair growth, mental or cardiorespiratory-metabolic function, and inflammatory/immune responses, leading to acute/chronic disorders [7].

### 1.3. Specific Effects of Stress on the Brain and the Cardiovascular and Immune Systems

Stress may exert a significant influence on various brain functions. More specifically, its role in memory is significantly dependent on the duration of exposure to the stress stimulus. Depending on the stress duration, memory can be either improved or worsened [8]. A specific-timed scheduled exposure to stress affects both hippocampus- and striatum-dependent memory. This further emphasizes the importance of stressful stimulus duration on memory function [9]. Additionally, the adverse effect of stress on cognition is dependent on the type, timing, intensity and duration of stress [10]. Generally, mild stress leads to improvement in cognitive function, especially regarding the virtual or verbal memory. However, if the stress intensity is above a predefined threshold, which varies between different individuals, it leads to cognitive disorders, mainly of memory/judgment, due to its effect on the hippocampus and prefrontal cortex [10].

The exposure to severe stress may trigger the development of malignancy through suppression of the immune system [8]. Furthermore, stress can decrease the activity of cytotoxic T lymphocytes and natural killer cells and promote the growth, genetic instability and tumor expansion of malignant cells [8]. Additionally, the post-stress high concentrations of norepinephrine have a negative effect on the immune function of phagocytes and lymphocytes [11]. Lastly, post-stress released catecholamines and opioids have immunosuppressive action [8].

Stress can be also involved in the function of the cardiovascular (CV) system. Stress-mediated risk factors with serious effects on the CV system include: (a) the increased stimulation of the sympathetic nervous system, (b) the induction of myocardial ischemia, (c) the development of supraventricular/ventricular arrhythmias, (d) the stimulation of platelet aggregation and (e) the development of endothelial dysfunction [12].

## 2. Magnetic Resonance Imaging of the Stress System

The noninvasive imaging assessment of the SS includes the evaluation of the brain, heart and immune system, as these are the main targets involved in the stress process that can be easily assessed using MRI.

### 2.1. Brain Magnetic Resonance Imaging (MRI) Evaluation

Although there has been significant progress in the histologic evaluation of brain, the revolution in the field of brain assessment during stress was achieved through MRI applications, such as diffusion (dMRI) and functional MRI (fMRI) [13].

#### 2.1.1. Diffusion MRI

**Figure 1:**
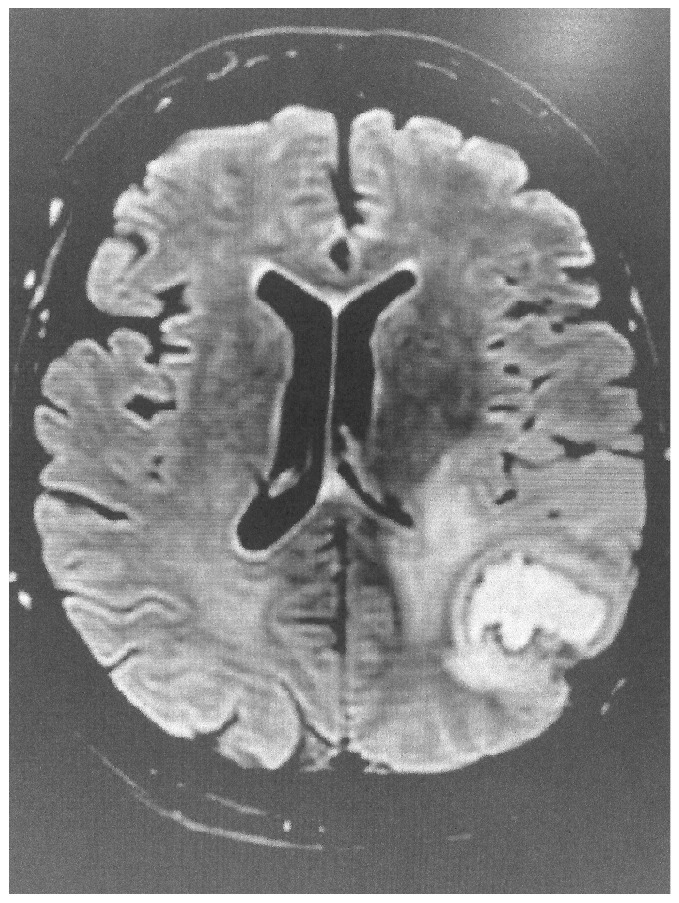
Brain FLAIR image showing evidence of stroke.

**Figure 2:**
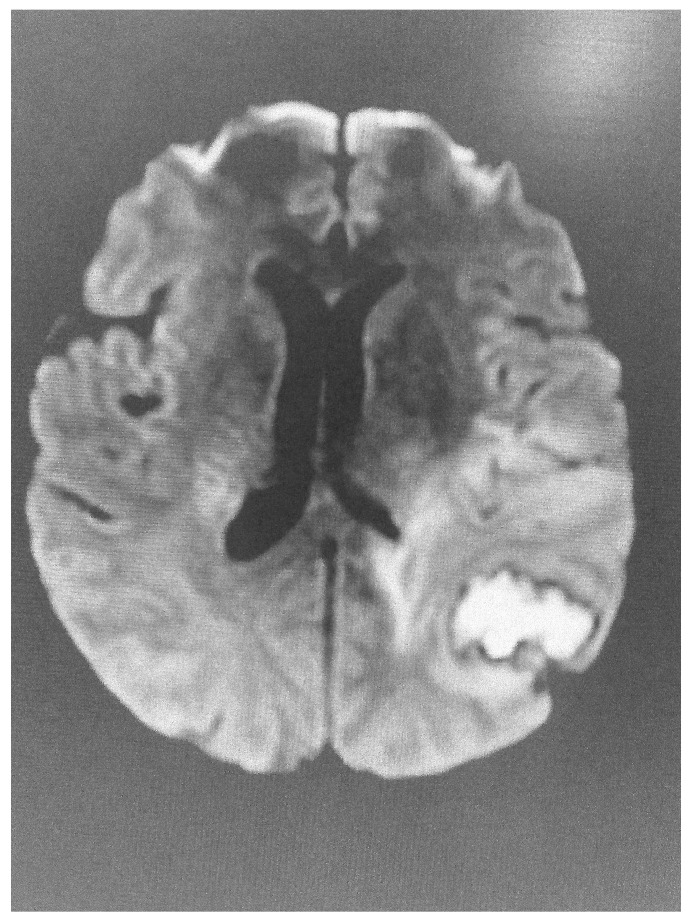
Abnormal diffusion image of the brain in the same patient, indicative of acute lesion.

dMRI utilizes the anisotropic water diffusion in white matter to produce micro-architectural details of white matter tracts and provide information about white matter integrity [14]. Through dMRI, we can define the structural connectivity between various brain areas and perform a detailed mapping of the connectional pathways of the brain [15]. White matter connectivity differences were identified using dMRI in military personnel exposed to trauma with or without post-traumatic stress disorder [16]. Furthermore, repeated overstimulation of the stress system, caused by prolonged exposure to highly stressful experiences, may affect brain structure, cognitive function and mental health. According to UK Biobank data, there is a clear link between stress and changes in brain microstructure, impairment in cognitive function and negative mental health outcomes [17]. Sex plays a distinct role in this reaction. Females and males present significant differences in white matter integrity when exposed to low levels of early life stress, with females showing lower white matter integrity compared with males. No difference in white matter integrity is observed in males and females exposed to mild stress. However, males are more sensitive to early life stress, while females are more sensitive if early life stress is followed by stress in adult life [18]. Diffusion images have been also used to clarify the acuity of lesions identified using FLAIR imaging (Figure 1 and Figure 2).

#### 2.1.2. Functional MRI

Functional magnetic resonance imaging (fMRI) can assess small changes in blood flow taking place during brain activity. It may be used for examining which parts of the brain are responsible for critical functions, for evaluating brain disorders and/or for guiding the treatment of brain disorders. fMRI allows detection of increased neural activity, either as increased local cerebral blood flow (CBF) or as changes in oxygenation concentration (Blood Oxygen Level Dependent, or BOLD contrast) [19]. Furthermore, resting state fMRI (rsfMRI) is the key tool for evaluation of the functional brain connectome at the time of not performing any explicit task [20,21].

fMRI has excellent spatial/temporal resolution and can assess changes of signal intensity due to blood oxygenation changes. Blood oxygenation levels vary according to neural activity and this variation can be used to evaluate the level of brain activity. An increased demand for energy/oxygen in the area of increased neural activity represents the basis of imaging using BOLD [22]. Recently, studies of the human brain using rsfMRI have revealed various areas presenting low-frequency, temporally correlated BOLD signal alterations [23]. Therefore, rsfMRI has become an important tool in the study of brain function even in the absence of overt behavioral changes [24].

#### 2.1.3. Limitations of fMRI

When discussing the drawbacks of fMRI, we should mention the potential presence of motion artifacts and the lack of precision that makes the interpretation of results difficult, as the areas that light up on MRI images may overlap between very different actions/feelings, making it hard to undertake a precise assessment of their clinical role. Lastly, fMRI is limited by its high cost and low availability [25]. However, clinical fMRI will advance beyond its current indications and move toward more diagnostic applications with the help of artificial intelligence (AI). AI enhances fMRI research by: (a) improving spatial/temporal resolution, (b) detecting subtle brain connectivity alterations and (c) supporting large-scale meta-analyses of data processing [26].

#### 2.1.4. Diffusion Versus Functional MRI

While dMRI utilizes the anisotropic water diffusion in white matter to detect details of white matter tracts [27], fMRI detects increased neural activity, either as increased CBF or as changes in oxygenation concentration (BOLD contrast) [28]. Additionally, arterial spin labeling (ASL), a noninvasive fMRI technique that uses arterial water as an endogenous tracer to measure CBF, provides reliable, absolute quantification of CBF with higher spatial/temporal resolution than BOLD [27], diffusion MRI (dMRI) and functional MRI (fMRI) [2].

Recent studies of the human brain using rsfMRI have detected various areas of low-frequency, temporally correlated BOLD signal fluctuations [23] even in the absence of clinically overt behavioral alterations [24]. Furthermore, the brain tended to be more integrated and less sensitive to acute stress, possibly in order to enable efficient stress-coping [27]. Additionally, memory function was stronger under stress owing to stress-induced cortisol response. Lastly, the amygdala/hippocampus connectivity during post-encoding awakened rest, regardless of stress or neutral situation, was associated with enhanced memory under stress [28].

To summarize, fMRI provides in vivo evaluation of central nervous system (CNS) alterations taking place during acute stress. Stress leads to activation of the Salience Network (SN), sensory hypersensitivity and parallel tasking. The organization and the functional connectivity (FC) of the distinct core neural networks involved in the stress response can be evaluated using different fMRI techniques, allowing the interpretation of brain functions at different timepoints. It is of particular interest that there is a progressive expansion of the brain “stress network” as part of normal child/adolescent development [28].

### 2.2. Cardiovascular Magnetic Resonance (CMR) Evaluation

#### 2.2.1. The Role of CMR in the Evaluation of Acute Myocardial Stress

The typical example of the effect of acute stress in the myocardium is Takotsubo Syndrome (TTS). This is characterized by acute, transient regional systolic dysfunction, dilatation, and oedema affecting mainly the apical and rarely the mid or basal part of the LV in the absence of obstructive coronary disease (CAD), although in small studies the presence of CAD was also identified [29]. Emotional or physical stress may lead to usually reversible HF. In the majority of cases, LV function returns to normal after a period of days or weeks. TTS is also known as stress cardiomyopathy (CM), but due to its transient nature, it was not included in the 2023 ESC Guidelines for CM [30,31].

CMR can visualize the typical regional wall motion abnormalities characteristic of TTS, such as apical or mid-ventricular ballooning. In addition, it can detect the presence of myocardial oedema and fibrosis (tissue characterization). The presence of oedema in the absence of positive late gadolinium enhancement (LGE), which represents replacement fibrosis, is typical of TTS. However, positive LGE can be found in patients with a mild cardiac enzyme elevation [3]. CMR may facilitate the differential diagnosis of other cardiac conditions, such as myocardial infarction, myocarditis, and ischemic and non-ischemic cardiomyopathies.

**Figure 3:**
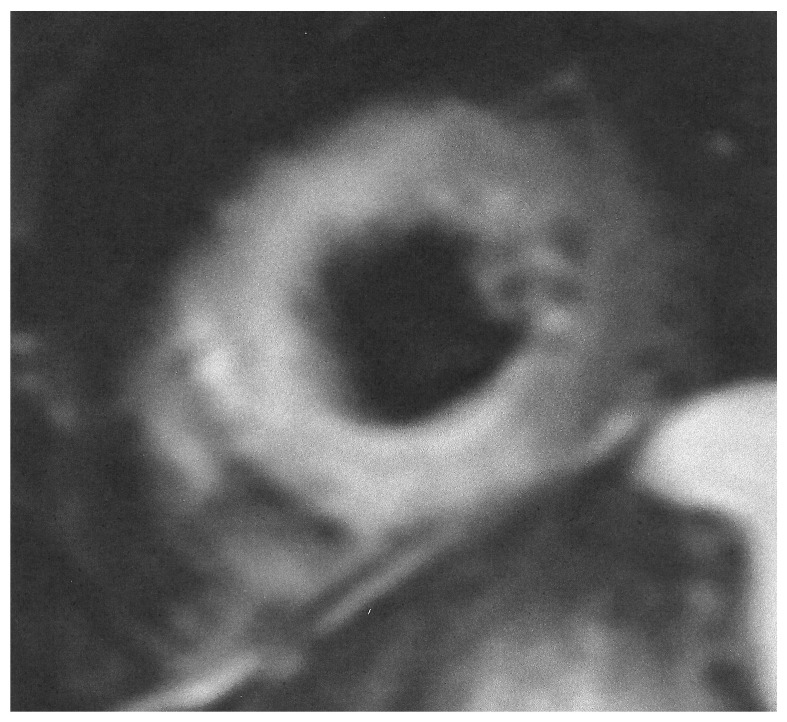
Short axis STIRT2 image showing extensive oedema in a patient with TTS.

**Figure 4:**
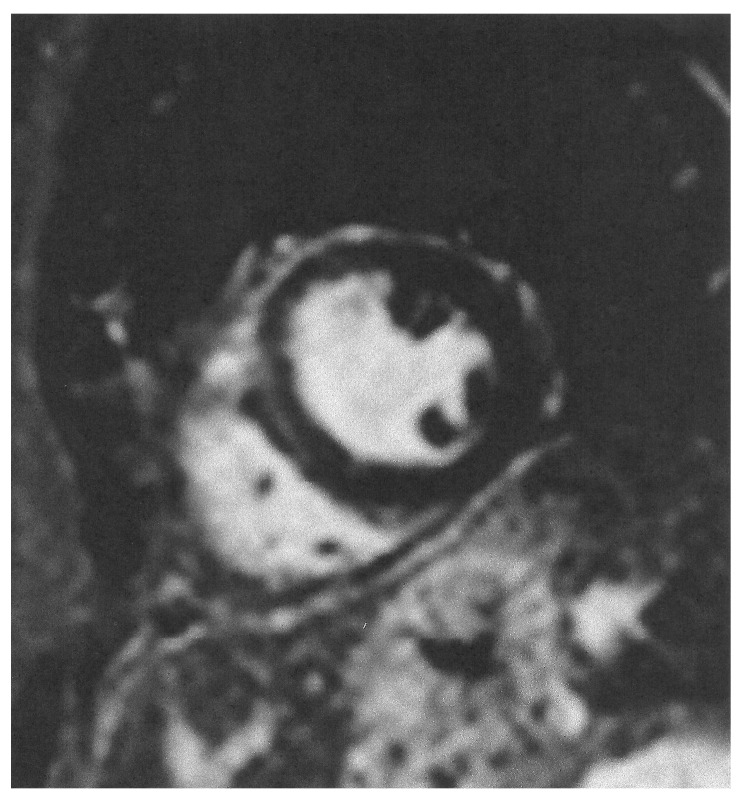
Short axis LGE image showing lack of replacement fibrosis in the same patient.

During the acute phase of TTS, CMR is recommended in doubtful cases, especially in those where different therapeutic protocols are needed, as in MINOCA or myocarditis. Furthermore, in the post-acute TTS phase, it is recommended in all patients within 2 months, if they have persisting ECG and/or regional wall motion abnormalities in the echocardiographic evaluation, in order to finally document the diagnosis of TTS [32]. CMR images of TTS are presented in Figure 3 and Figure 4.

#### 2.2.2. The Role of CMR in the Evaluation of Chronic Myocardial Stress

The main causative factor of chronic myocardial stress is myocardial ischemia. This can occur due to (a) mental stress, (b) conventional stress and/or (c) the combination of both. Vaccarino et al. presented the largest (*n* = 918) and most diverse (34% women, 40% Black individuals) observational study regarding the association between mental stress ischemia, conventional stress ischemia and future cardiac events in patients with known CAD using SPECT imaging. Overall, mental stress ischemia was observed in 16% of participants, conventional stress ischemia in 31% and both in 10%. After a median 5-year follow-up, the primary end point of CV death or myocardial infarction occurred in 17% of the participants, and a secondary end point, including hospital admission due to HF, occurred in 35%. In all models tested, the presence of both mental and conventional stress ischemia showed significant associations with both the primary and secondary end points. However, analyses based on the specific ischemia phenotype demonstrated that participants with both phenotypes had the strongest association with the primary end point, followed by individuals with mental stress ischemia only [33].

From the pathophysiologic point of view, mental stress ischemia and conventional stress ischemia appear to be triggered by different mechanisms. Mental stress may lead to an adrenergic overactivity, but in contrast to exercise-induced ischemia, mental stress ischemia typically appears at a lower cardiac work-load and therefore it is not associated with the severity of CAD [33]. Moreover, mental stress ischemia is independent of hemodynamic changes, showing that myocardial oxygen demand and coronary artery lesions play a relatively minor role in mental ischemia. Furthermore, invasive studies have shown that in mental stress ischemia, coronary blood flow decreases without significant change of the coronary diameter at the site of atherosclerotic lesion, suggesting a dysfunction of the coronary microcirculation. Furthermore, patients with mental stress ischemia also have an impaired vasodilatory response to intracoronary acetylcholine infusion, in parallel with coronary endothelial dysfunction [34,35]. In contrast to mental stress, the conventional stress induced using vasodilators, such as dipyridamol, adenosine or regadenoson, increases coronary blood flow through endothelium-independent mechanisms [36], while classic exercise does so mainly through endothelium-dependent changes in coronary flow [36].

Currently, stress CMR has been proposed as the ideal noninvasive test that can cost-effectively and accurately evaluate myocardial ischemia/viability and cardiac function without the use of ionizing radiation. There is an abundance of randomized controlled trials validating its diagnostic performance, risk stratification capabilities and ability to guide the use of coronary interventions. More specifically, stress CMR has shown higher diagnostic sensitivity than single-photon emission computed tomography (SPECT) imaging in detecting angiographically significant CAD. It is of great value for risk stratification of patients with moderate to high pretest probability of stable ischemic heart disease and for patients with known challenging imaging findings, including women, patients with prior revascularization and those with left ventricular dysfunction. The currently existing data support its routine clinical application, the additive value of myocardial blood flow quantification and the simultaneous evaluation of myocardial function/viability that is routinely performed in all CAD patients [36,37,38].

#### 2.2.3. OS-CMR as an Index of Vascular Function in Acute/Chronic Cardiac Disease

Oxygenation-sensitive cardiac magnetic resonance imaging (OS-CMR) is a diagnostic technique that uses the inherent paramagnetic properties of deoxyhemoglobin as an endogenous factor of tissue contrast. If it is combined with a protocol of standardized vasoactive breathing maneuvers that includes a combination of hyperventilation and apnea as a “nonpharmacologic vasomotor stimulus”, OS-CMR can identify changes in myocardial oxygenation. The quantification of changes during the cardiac cycle and through the vasoactive maneuvers can provide indices of coronary macro-/micro-vascular function and therefore it may overcome the need for any extrinsic infusion of contrast and/or pharmacologic stress agents [39].

OS-CMR is based on the sensitivity of T2*-weighted images to detect changes in blood oxygenation and can be acquired on any cardiac MRI scanner using a modified standard clinical steady-state free precession (SSFP) cine sequence, making this technique easily applicable in clinical practice. The vasoactive breathing maneuver includes the application of a 4 min breathing protocol with 120 s of free breathing and 60 s of paced hyperventilation, followed by an expiratory breath-hold of at least 30 s. The regional and global response of myocardial oxygenation to this maneuver can be assessed by evaluation of the signal intensity changes. The change over the initial 30 s of the post-hyperventilation breath-hold, referred to as the breathing-induced myocardial oxygenation reserve, has been already evaluated in healthy people and various pathologies [40]. The application of OS-CMR has no extra cost, as the sequence used is widely available in 3T MRI systems. However, training in specific software for data analysis could result in an additive cost.

### 2.3. Immune System Assessment Using MRI

#### 2.3.1. Combined Brain-Immune System MRI Assessment

Although the blood–brain barrier (BBB) was thought to protect the brain from the effects of the immune system, immune cells can migrate from the blood to the brain, due to central nervous system (CNS) diseases, influencing both disease evolution and prognosis. Furthermore, the spleen, which is an important immune system organ, acts as a filter for blood within the immune system. There is increasing evidence supporting the hypothesis that the spleen affects various brain functions via immune modulation. Systemic inflammation or chronic social defeat stress (CSDS) can cause splenomegaly in rodents. Importantly, the new antidepressant arketamine could normalize splenomegaly and depression-like behaviors in CSDS-susceptible mice. A recent study supports the direct connection between brain and spleen [41,42,43,44]. The spleen can also regulate the humoral immune response through two brain regions including corticotropin-related neurons in the paraventricular nucleus (PVN) and central nucleus of the amygdala (CeA). Furthermore, afferent and efferent vagus nerve signaling may contribute to brain/spleen communication [41].

Iron oxide nanoparticles have been extensively used for diagnosis and treatment assessment in various inflammatory diseases. Depending on their core size, iron oxide nanoparticles can be either used as T1-weighted or T2/T2*-weighted MR contrast agents. Ferucarbotran and ferumoxide have been used for visualization of liver tumors and metastases. Ferumoxsil is approved by the FDA as an orally administered contrast agent for gastrointestinal imaging. Ferumoxtran and ferumoxytol are in clinical trials for vascular imaging and lymph node metastasis assessment [42].

Signal loss observed in the brain after the administration of ultrasmall super-paramagnetic particles of iron oxide (USPIO) has been correlated with immune cell activity in inflammatory areas in patients with multiple sclerosis. Uptake of USPIO by circulating monocytes and their migration towards inflammatory areas have been considered as the most important mechanism for USPIO uptake by brain parenchyma. MR images of cervical lymph nodes showed USPIO accumulation at 24 h after administration that stabilized at 72 h. Histologic analysis revealed that USPIO accumulated in infiltrated macrophages in the medulla and subcapsular sinus, entering the central nervous system directly post-administration. This indicates the involvement of a damaged blood–brain barrier in USPIO-associated MR alterations [43].

#### 2.3.2. Combined Heart and Immune System MRI Assessment

Ferumoxytol is the only USPIO clinically available in the U.S.A and has a potential use in the detection of macrophage-infiltrated cardiovascular tissues. As an iron supplement, it was approved for treatment of iron deficiency anemia. The iron core of ferumoxytol is incorporated into the body, once it is phagocytosed by macrophages. In tissues with high inflammatory cell concentration, such as atherosclerotic plaques and myocardial infarction, localization of iron-loaded macrophages can be visualized on delayed MRI images. The iron core of ferumoxytol leads to a shortening of T2*/T2 relaxation rates. Areas with high macrophage concentration appear hypointense (negative contrast) on T2/T2* MRI. Recently, in vitro findings have supported the potential specificity of ferumoxytol interactions with macrophage subtypes, which may have implications for therapeutic interventions. With increasing concerns about gadolinium retention in the brain and other tissues, the positive safety profile of ferumoxytol-enhanced MR is of great value [44].

USPIOs were identified in both myocardial macrophages and myocardial interstitium. The R1 time-course reflected passive interstitial distribution whereas the multi-time-point R2* was also sensitive to active macrophage uptake. The R2*/R1 ratio provided a quantitative measurement of myocardial macrophage infiltration. An R2*/R1 threshold of 25 had a sensitivity and specificity of 90% and 83%, respectively, for detecting active USPIO uptake [45]. However, in patients with acute myocarditis, USPIO-enhanced MRI did not provide additional clinically useful information compared with modified LLC, suggesting that the detection of tissue macrophages does not play an important role in myocarditis [46].

More specifically, regarding the acute myocardial stress evaluation, a study of patients with TTS CM, who underwent multiparametric CMR using USPIO for detection of myocardial macrophages, showed that TTS CM was characterized by a myocardial macrophage inflammatory infiltrate, changes in the distribution of monocyte subsets and an increase in systemic pro-inflammatory cytokines that persisted for at least 5 months, suggesting a low-grade chronic inflammation [47].

Another promising approach in the evaluation of myocardial immune cells is Fluorine-19 magnetic resonance spectroscopy (19F-MRS). 19F-MRS is a unique cardiovascular imaging technique with several characteristics that can provide information complementary to other imaging modalities. In the preclinical setting, it has been successfully used to quantify and monitor inflammation in several diseases [48].

Currently, we can directly image the ^19^F nucleus instead of the hydrogen (^1^H) in water. Since there is no naturally detectable ^19^F in the body, an injected tracer will generate a directly proportional signal. These tracers are tailored nanoparticles filled with perfluorocarbons (PFC), which are safe and inert molecules that are readily taken up by circulating immune cells. This uptake and the proportional signal enable the direct quantification and monitoring of inflammation over time with exceptional specificity even at low cell concentrations. These advantages together help us gain a deeper understanding of inflammatory processes within the myocardium.

Currently, the most feasible target for a first clinical trial is either (a) acute myocardial infarction (AMI), which has a high concentration of leukocytes and a direct link of the inflammation load to myocardial function, or (b) carotid atherosclerosis, where the inflamed plaque is close to the surface and can present high signals, while a gold-standard comparison can be made through histology of the excised plaque after endarterectomy [48].

#### 2.3.3. Combined Brain-Heart MRI Assessment

This approach can illustrate brain–heart interaction, by providing combined information about the brain and the heart in the same examination. Our group has already published results about combined brain-heart imaging in patients with various autoimmune rheumatic diseases [49,50]. Therefore, we propose this approach as a comprehensive assessment of brain–heart interaction in pathologic entities involving both organs, such as TTS and traumatic brain injury [3,51]. However, for the evaluation of the stress system a more detailed analysis, including brain–heart–immune system interaction post mental/conventional stress application, is needed.

##### Current Limitations of Imaging of the Stress System

Although the evaluation of SS imaging is extremely promising for individualization of patients’ risk stratification and treatment, it also has important limitations including the following issues.

1. Availability. There is great variation in scanners availability between continents and countries.
2. Cost. There is great variation in cost and insurance cover between countries, and studies regarding a combined BHI MRI are currently not available.
3. Some more sophisticated MRI techniques, such as the application of ferumoxytol, have not been approved in some countries in Europe.
4. 19F MRI is currently approved only for animal studies.
5. Software for evaluation of stress CMR and OS-CMR is not widely available.
6. MRI personnel need further training to be able to perform the combined BHI evaluation.

## 3. Hair Cortisol and Brain–Heart—Immune System Function During Stress

The analysis of hair cortisol concentrations (HCCs) is a relatively new strategy to measure long term cumulative cortisol levels, which is increasingly used in psycho-neuro-endocrinologic research and represents the most easy and simple way to assess the stress system [52]. Hair cortisol provides a quantification of total cortisol secreted into hair over several weeks. There is increasing evidence suggesting that elevated hair cortisol levels are associated with both the incidence of CVD and the treatment outcomes. Moreover, increased hair cortisol concentration has been linked with established cardiometabolic risk factors for CVD, including high blood pressure, diabetes and adiposity. Hair cortisol is a reliable biomarker of chronic cortisol excess, which may contribute to pathogenesis/prognosis of CVD. However, the current evidence relies on small-scale cross-sectional studies [53,54]. Furthermore, hair cortisol levels were positively associated with BMI, WC, IL-6 and leukocyte numbers in cross-sectional analyses and with increases in BMI and WC in longitudinal analyses. Although studies about causality are still missing, higher long-term glucocorticoid levels may represent a risk factor for the development of obesity [55].

We should also note that white matter microstructure in 58 children, aged 5–9 years after exposure to stressful life conditions, were associated with higher hair cortisol concentrations. Furthermore, physiological stress, identified by hair cortisol, was associated with higher fractional anisotropy in the cingulum bundle [56]. Regarding the heart, HCCs were significantly high in patients with angiographically confirmed coronary atherosclerosis compared with controls and had a significant positive correlation with diabetes and obesity [57]. Lastly, stressful life events trigger immunological alterations that persist across time and promote a continuous effect on white blood cell distribution that might induce subclinical inflammatory reaction and depression of the immune system and may finally act as a link between psychological stress and physical disease [58].

It seems that hair cortisol can be of value as an initial, reliable stress index of the brain and heart. However, it is an “all or nothing index” that cannot provide detailed information regarding preclinical alterations taking place in the brain–heart–immune system axis during various types of stress. In contrast, the high spatial resolution and the multifaceted ability of MRI can provide clinically useful details about the quantification and evolution of stress in each system/organ.

## 4. The Stress System in Heart Failure

CVD represents a serious worldwide health problem, with increasing evidence of differences between women and men regarding epidemiology, pathophysiology, clinical management and future cardiac events. Literature data suggest that women experience a doubled incidence of CVD-related deaths, while angina, HF and stroke are highly prevalent in females. About 20–25% of women suffer from depression during their life, and depressive symptoms have been considered as an emergent, non-traditional risk factor for CVD in the female general population.

Underlying mechanisms explaining the link between depression and CVD may range from behavioral to biological risk factors, including sympathetic nervous system hyperactivity and impairment in hypothalamic–pituitary–adrenal function. However, the neuroendocrine background can only partially explain the above-discussed differences for chronic systemic inflammation, altered hemostasis and modulation of cardiac autonomic control. In addition, there is evidence suggesting the presence of gender differences in biological responses to mental stress [59].

Taking under consideration the complex involvement of the brain in the development of acute/chronic heart disease and finally HF, it is clear that the evaluation of the interaction between the brain and heart is of great pathophysiologic significance. These thoughts have been recently presented in a position paper from the Study Group on Heart and Brain Interaction of the Heart Failure Association [60].

In addition, it has been shown that inflammation plays a significant role in the pathophysiology of HF. Furthermore, the maladaptive responses of both the innate and the adaptive immune system contribute to adverse cardiac remodeling, fibrosis, and vascular and extra-cardiac organ dysfunction. Multiple mechanisms are involved in this process that still remain poorly understood. However, it is clear that any improvement in inflammatory pathways may lead to clinical amelioration of HF, in parallel with the development of novel therapies targeting HF. Biomarkers may also have a place in the identification of patients that may benefit from the optimization of HF therapy. The above findings make necessary the incorporation of immune system evaluation during HF [61].

## 5. MRI of the Stress System. Moving Toward a Revolutionary Holistic Approach

The interaction between the brain, heart and immune system has been already documented in various stress situations. CV complications are common at the time of post traumatic brain injury (TBI) leading to increased morbidity/mortality. This is due to immune cell infiltration/inflammation in the heart with concurrent cardiac dysfunction that TBI induces. It has also been proven that splenectomy can improve cardiac inflammation and function after TBI [51]. Similarly, splenectomy attenuates the intracerebral hemorrhage (ICH)-induced neurological/cognitive impairment as well as ICH-induced cardiac dysfunction in mice. Inflammatory cell infiltration into heart and immune responses mediated by the spleen may contribute to ICH-induced acute/chronic cardiac dysfunction [52].

The above findings clearly describe the interaction between the brain, heart and immune system during acute/chronic stress and emphasize the need for a holistic imaging approach to the SS. However, potential questions could be raised regarding the following topics:

(a) What is the reason for such an approach?
(b) Is this approach cost-effective?
(c) What is the clinical benefit of this combined evaluation?
(d) Can we individualize treatment based on a combined evaluation?
(e) What is the additive value of imaging in comparison with hair cortisol and other routinely used clinical tests?

Currently, we do not know how stress influences the intensity of the brain–heart–immune system reaction. Additionally, we never evaluated the reaction of each system separately and did not apply individualized, tailored treatment according to the effect of stress in various tissues. We believe that this holistic approach will improve our knowledge about stress mechanisms and facilitate a more effective, individualized treatment. However, in order to achieve this target, we need a robust noninvasive imaging tool among other indices. As MRI is a noninvasive modality without radiation, it seems ideal for this target. Therefore, we suggest the following protocol:

(a) Brain evaluation using FLAIR, T1-W imaging before and after contrast and functional MRI to assess brain function during any kind of acute/chronic CVD.
(b) Cardiac Imaging using SSFP for function assessment, LGE for replacement fibrosis, T2 for oedema imaging, T1 and ECV mapping for diffuse fibrosis, and OS-CMR using breathing maneuvers for early detection of vascular changes during any kind of acute/chronic CVD.
(c) Immune system assessment using USPIO and 31-F during any kind of acute/chronic CVD.
(d) Hair cortisol measurement in cases of chronic stressful situation involving the CV system.

All this information may finally lead to a diagnostic algorithm regarding imaging the stress system in acute/chronic CVD. Proposing such an algorithm is currently not feasible, due to lack of necessary evidence. However, the above-mentioned arguments regarding the role of stress in CVD deserve further intense evaluation in various CVDs.

A currently available algorithm for evaluation of the SS should include:

(a) Evaluation of hair cortisol as a first step to assess the stress status of the patient.
(b) Brain MRI using dMRI and, if available, fMRI.
(c) CMR using SSFP sequences for function, T1 perfusion imaging for stress myocardial perfusion/fibrosis, T2 mapping for oedema assessment and nat T1 mapping and ECV for micro-fibrosis assessment.
(d) Evaluation of USPIO-enhanced MRI for immune system assessment.

However, more sophisticated approaches should be moved gradually from research to clinical routine.

## 6. Conclusions

The brain-immune system-heart axis plays an important role in the pathogenesis and prognosis of various heart diseases including HF, and stress plays a pivotal role in this interaction. MRI is the ideal noninvasive tool without radiation that can provide valuable information about these structures using targeted protocols. Although we do not know how stress influences the intensity of the brain–immune system-heart-axis, a holistic approach using MRI has the potential to augment our understanding of this highly complex and multilevel understanding, potentially leading to advances in diagnosis and treatment of various disorders. However, a multidisciplinary approach is mandated due to the multi-organ nature of this interaction, to promote further scientific advancements in this field.

## 7. Take Home Messages

1. Stress plays a crucial role in the development and evolution of acute/chronic CVD.
2. Hair cortisol is an initial, reliable stress index of brain and heart involvement.
3. Heart–brain–immune system axis activation during acute/chronic cardiac disease is important for survival.
4. MRI is a noninvasive tool that can image the stress system effect on the heart–brain–immune system without radiation and allows for an individual assessment of the effect of stress on each system.
5. Although an algorithm regarding “when and how” we can apply imaging of the stress system is currently not available, there are strong data supporting that such an endeavor will significantly contribute to our efforts for personalized medicine.

## Data Availability

No new data were created or analyzed in this study. Data sharing is not applicable to this article.
